# BLAST+: architecture and applications

**DOI:** 10.1186/1471-2105-10-421

**Published:** 2009-12-15

**Authors:** Christiam Camacho, George Coulouris, Vahram Avagyan, Ning Ma, Jason Papadopoulos, Kevin Bealer, Thomas L Madden

**Affiliations:** 1 National Center for Biotechnology Information, National Library of Medicine, National Institutes of Health, Building 38A, 8600 Rockville Pike, Bethesda, MD 20894, USA

## Abstract

**Background:**

Sequence similarity searching is a very important bioinformatics task. While Basic Local Alignment Search Tool (BLAST) outperforms exact methods through its use of heuristics, the speed of the current BLAST software is suboptimal for very long queries or database sequences. There are also some shortcomings in the user-interface of the current command-line applications.

**Results:**

We describe features and improvements of rewritten BLAST software and introduce new command-line applications. Long query sequences are broken into chunks for processing, in some cases leading to dramatically shorter run times. For long database sequences, it is possible to retrieve only the relevant parts of the sequence, reducing CPU time and memory usage for searches of short queries against databases of contigs or chromosomes. The program can now retrieve masking information for database sequences from the BLAST databases. A new modular software library can now access subject sequence data from arbitrary data sources. We introduce several new features, including strategy files that allow a user to save and reuse their favorite set of options. The strategy files can be uploaded to and downloaded from the NCBI BLAST web site.

**Conclusion:**

The new BLAST command-line applications, compared to the current BLAST tools, demonstrate substantial speed improvements for long queries as well as chromosome length database sequences. We have also improved the user interface of the command-line applications.

## Background

Basic Local Alignment Search Tool (BLAST) [1,2] is a sequence similarity search program that can be used to quickly search a sequence database for matches to a query sequence. Several variants of BLAST exist to compare all combinations of nucleotide or protein queries against a nucleotide or protein database. In addition to performing alignments, BLAST provides an "expect" value, statistical information about the significance of each alignment.

BLAST is one of the more popular bioinformatics tools. Researchers use command-line applications to perform searches locally, often searching custom databases and performing searches in bulk, possibly distributing the searches on their own computer cluster. The current BLAST command-line applications (i.e., blastall and blastpgp) were available to the public in late 1997. They are part of the NCBI C toolkit [3] and are supported on a number of platforms that currently includes Linux, various flavors of UNIX (including Mac OS X), and Microsoft Windows.

The initial BLAST applications from 1997 lacked many features that are presently taken for granted. Within three years of the initial public release, BLAST was modified to handle databases with more than 2 billion letters, to limit a search by a list of GenInfo Identifiers (GIs), and to simultaneously search multiple databases. PHI-BLAST [4], IMPALA [5], and composition-based statistics [6] were also introduced within this time period, followed by MegaBLAST [7] and the concept of query-concatenation (whereby the database is scanned once for many queries). Chris Joerg of Compaq Computer Corporation suggested performance enhancements in 1999. A group at Apple, Inc. suggested other enhancements in 2002 [8]. These and other features were of great importance to BLAST users, but the continual addition of unforeseen modifications made the BLAST code fragile and difficult to maintain.

Many mammalian genomes contain a large fraction of interspersed repeats, with 38.5% of the mouse genome and 46% of the human genome reported as interspersed repeats [9]. Traditionally, the only supported method available to mask interspersed repeats in stand-alone BLAST has been to execute a separate tool (e.g., RepeatMasker [10]) on a query, produce a FASTA file with the masked region in lower-case letters, and have BLAST treat the lower-case letters as masked query sequence. This requires separate processing on each query before the BLAST search.

NCBI recently redesigned the BLAST web site [11] to improve usability [12], which helped to identify issues that might also occur in the stand-alone BLAST command-line applications. These changes have, unfortunately, made it more difficult to match parameters used in a stand-alone search with default parameters on the NCBI web site.

The advent of complete genomes resulted in much longer query and subject sequences, leading to new challenges that the current framework cannot handle. At the same time, increases in generally available computer memory made other approaches to similarity searching viable. BLAT [13] uses an index stored in memory. Cameron and collaborators designed a "cache-conscious" implementation of the initial word finding module of BLAST [14]. The concerns listed in this section and the start of a new C++ toolkit at the NCBI [15] motivated us to rewrite the BLAST code and release a completely new set of command-line applications. Here we report on the design of the new BLAST code, the resulting improvements, and a new set of BLAST command-line applications.

In this article, a search type is described by a word or two in all upper-case letters. For example, a BLASTX search translates the nucleotide query in six frames and compares it to a protein database.

## Implementation

This section reports first on the overall design of the new software and then discusses several enhancements to BLAST.

### Overall design

Two criteria were most important in the design of the new BLAST code: 1.) the code structure should be modular enough to allow easy modification; and 2.) the same BLAST code should be embedded in at least two different host toolkits. This would allow both the new NCBI C++ toolkit and the older NCBI C toolkit to use the same BLAST source code.

**Figure 1:**
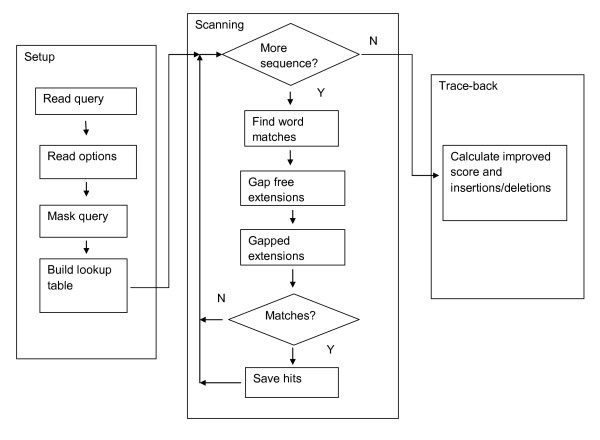
**Schematic of a BLAST search**. The first phase is "setup". The query is read, low-complexity or other filtering might be applied to the query, and a "lookup" table is built. The next phase is "scanning". Each subject sequence is scanned for words ("hits") matching those in the lookup table. These hits are further processed, extended by gap-free and gapped alignments, and scored. Significant "preliminary" matches are saved for further processing. The final phase in the BLAST algorithm, called the "trace-back", finds the locations of insertions and deletions for alignments saved in the scanning phase.

At a high level, the BLAST process can be broken down into three modules (Figure 1). The "setup" module sets up the search. The "scanning" module scans each subject sequence for word matches and extends them. The "trace-back" module produces a full gapped alignment with insertions and deletions.

The setup phase reads the query sequence, applies low-complexity or other filtering to it, and builds a "lookup" table (i.e., perfect hashing). The lookup table contains only words from the query for nucleotide-nucleotide searches such as BLASTN or MEGABLAST. DISCONTIGUOUS MEGABLAST allows non-consecutive matches in the initial seed. Protein-protein searches such as BLASTP allow "neighboring" words. The neighboring words are similar to a word in the query, as judged by the scoring matrix and a threshold value.

The scanning phase scans the database and performs extensions. Each subject sequence is scanned for words ("hits") matching those in the lookup table. These hits are used to initiate a gap-free alignment. Gap-free alignments that exceed a threshold score then initiate a gapped alignment, and those gapped alignments that exceed another threshold score are saved as "preliminary" matches for further processing. The scanning phase employs a few optimizations. The gapped alignment returns only the score and extent of the alignment. The number and position of insertions, deletions and matching letters are not stored (no "trace-back), reducing the CPU time and memory demands. Searches against nucleotide subject sequences consider only unambiguous bases (A, C, G, T), with ambiguous bases (e.g., N) replaced at random during preparation of the BLAST database or subject sequence. A four letter alphabet allows packing of four bases into one byte, and the subject sequences are scanned four letters at a time. Finally, less sensitive heuristic parameters are employed for the gapped alignment, and the full extent of a gapped alignment may, in rare cases, not be found.

The final phase of the BLAST search is the trace-back. Insertions and deletions are calculated for the alignments found in the scanning phase. Ambiguous bases are restored for nucleotide subject sequences, and more sensitive heuristic parameters are used for the gapped alignment. Composition-based statistics [6] may also be applied for BLASTP (protein-protein) and TBLASTN (protein compared against translated nucleotide subject sequences).

Ideally, one should be able to independently replace the functionality described in each of the small rectangles of Figure 1 (e.g., "build lookup table") with another implementation. Some coordination is required: for example, the lookup table is used when finding word matches, so both "build lookup table" and "find word matches" need to be changed together. Finding word matches is the most computationally intensive part of the BLAST search, so the implementation should be as fast as possible. To address this, the author of the lookup table implementation must provide the scanning routine for finding word hits. Other modules can be changed independently.

The selection of ISO C99 allows use of the new BLAST code in both C and C++ environments. The host toolkit provides a software layer to allow BLAST to communicate with the rest of each toolkit. This design requires a clean separation between the algorithmic part of BLAST and the module that retrieves subject sequences from the database. To allow this, the retrieval of subject sequences for processing by the core of the BLAST code is performed through an Abstract Data Type (ADT), which specifies a set of data values and permitted operations. The actual retrieval occurs through an implementation of the ADT in the host toolkit. The implementation can be changed depending upon the need and requires no changes to the BLAST algorithm code itself.

The subject sequence information required by BLAST is quite simple. It consists of the total number of sequences to be searched, the length of any given sequence, as well as methods to retrieve the actual sequence. The total database length is needed for calculation of expect values. A database name and the length of the longest subject sequence are also required to implement some functions in an efficient manner. In order to satisfy the above requirements, an ADT, called the BlastSeqSrc [16], was implemented.

### Database masking

Low-complexity regions and interspersed repeats typically match many sequences. These matches are normally not of biological interest, may lead to spurious results, and confound the statistics used by BLAST. BLAST offers two query masking modes to avoid such matches. One is known as "hard-masking" and replaces the masked portion of the query by X's or N's for all phases of the search. On the other hand, "soft-masking" makes the masked portion of the query unavailable for finding the initial word hits, but the masked portion is available for the gap-free and gapped extensions once an initial word hit has been found.

The BLAST databases can also be masked. Masking information is stored as a series of intervals, so that masking can be switched on or off. Information from multiple masking algorithms can be stored in the same BLAST database and accessed separately. Currently, database masking consists of skipping masked portions of the database during the scanning phase, but it is still possible to extend through masked portions of the database; as such, database masking is analogous to soft-masking a query.

### Minimizing memory and cache footprint

Modifications that reduce the CPU time and memory footprint of BLAST searches with long query or subject sequences are examined. First, an optimization for the scanning phase of the BLAST search is presented. Then, an improvement for the trace-back phase is described.

BLAST searches with very large queries are routine, but some of the data structures scale with the query length. The following analysis examines the scanning phase (Figure 1) of the BLAST search.

Two large structures are frequently accessed during the scanning phase. The first is the "lookup table", which maps words in a subject sequence to positions in the query. The second is the "diag-array", which tracks how far BLAST has already extended word hits on any given diagonal; its size scales with the query length. The scanning phase is a large fraction of the time of most BLAST searches, so these structures must be accessed quickly. Contemporary CPUs typically communicate with main memory through several levels of cache, called a "memory hierarchy". For example, the L1 cache is the smallest and has the lowest latency; the L2 cache is larger but slower. On a machine with an Intel Xeon CPU, the L1 cache might be around 16 kB and the L2 cache can range in size from 0.5-4 MB. If the CPU does not find data or an instruction in the cache, it must fetch it from main memory; a "cache miss". Performance could be improved by making the lookup table and diag-array small enough to fit into L2 cache, still leaving room for instructions and other data.

In order to be specific, the discussion in the next two paragraphs is limited to a BLASTX search, which translates a nucleotide query in six frames (three frames on each strand) and compares it to a protein database.

The lookup table contains a long array (the "backbone"), with each cell mapping to a unique word. The lookup table translates each residue type to a number between 1 and 24, so a three-letter word maps to an integer between 1 and 24^3^. For a three-letter word, an array of 32768 (32^3^) cells allows a quick calculation of the offset into the backbone while scanning the database for word matches. Each cell of the backbone consists of four integers. The first integer specifies how many times that word appears in the query; the other three can have one of two functions. For three or fewer occurrences, the three integers simply specify the positions of the word in the query. If there are more than three occurrences, however, the integers are an index into another array containing the positions of the word in the query. The total memory occupied by the backbone is 16 bytes × 32768, or about 524 kB. Finally, there is a bit vector occupying 4096 bytes (32768/8). The corresponding bit is set in the bit vector for backbone cells containing entries. For a short query, where the backbone may be sparsely populated, this allows a quick check whether a cell contains any information.

A BLASTX query of N nucleotides becomes twice as long when it is represented as six protein sequences. The diag-array consumes one four-byte integer per letter in the query. An estimate of the total memory occupied by the lookup table backbone and the diag-array, in bytes, for a nucleotide query of length N is:

For a query of N = 50 k, this is close to a million bytes, already the total size of L2 cache in many computers used for BLAST searching. Modifications to these structures might permit larger queries, but for contigs and chromosomes the structures would still overflow the L2 cache. To overcome this, the query is split into smaller overlapping pieces for the scanning phase of the search. BLAST then merges the results and aligns the entire query during the trace-back phase, obtaining the same results as a search that was not split. Splitting the query has an additional advantage; since the sub-query used during the scanning phase is of bounded length, it is possible to use a smaller data type in the lookup table (specifically, a two byte rather than a four byte integer). This reduces the first term in the above equation from 528,384 to 266,240 bytes.

The final phase of the BLAST search, the trace-back, processes the preliminary matches, producing an alignment with insertions and deletions. Additionally, heuristic parameters may be assigned a more sensitive value, ambiguities in a nucleotide database sequence are resolved, and the composition of the subject sequences may be taken into account when calculating expect values. Some subject sequences must be retrieved again for this calculation, but since the preliminary phase finds the rough extent of any alignment, the entire sequence is often not needed. This is most important for short queries searched against a database of much longer sequences. Only part of the subject sequences, when appropriate, is now retrieved, and performance results are presented under "Partial subject sequence retrieval" below.

## Results and discussion

First, we introduce a set of BLAST command-line applications built with the software library discussed above. Then, we present an example use of database masking as well as two performance analyses that demonstrate improvements in search time: searches with very long queries and searches of chromosome-sized database sequences. For each performance analysis, we prepared a baseline application that disables the new feature being tested. Finally, we discuss an example of retrieving subject sequences from an arbitrary source.

A SUSE Linux machine with an Intel Xeon 3.6 GHz CPU, 16 kB of L1 cache, 1 MB of L2 cache, and 8 GB of RAM, provided data for the comparisons described here.

### BLAST+ command-line applications

New command-line applications have been developed using the NCBI C++ toolkit, and they are referred to as the BLAST+ command-line applications (or BLAST+ applications). Extensive documentation about the different command-line options is available [17], so only general comments about the interface are presented here. The NCBI C++ toolkit argument parser permitted the use of multi-letter command-line arguments. New BLAST+ command-line applications were introduced, dependent upon the molecule types of the query and subject sequences. For example, there is a "blastx" application that translates a nucleotide query and compares it to a protein database, and a "blastn" application that compares a nucleotide query to a nucleotide database. The command-line options and help messages are specific to each application. In contrast, the current C toolkit command-line application ("blastall") presents usage instructions about nucleotide match and mismatch scores, needed only for BLASTN, even if the user wants to perform a BLASTX search. Users also need to optimize for different tasks within a single command-line application. For example, MEGABLAST compares a nucleotide query to a nucleotide database, but is optimized for closely related sequences (e.g., searching for sequencing errors), using a large word size and a linear gap penalty. BLASTN, on the other hand, is the traditional nucleotide-nucleotide search program and uses a smaller word size and affine gapping by default. The concept of a "task" allows a user to optimize the search for different scenarios within one application. Setting the task for the blastn application changes the default value of a number of command-line arguments, such as the word size, but also the default scoring parameters for insertions, deletions, and mismatches. These values are changed to typical values that would be used with the selected task. For the MEGABLAST task, the nucleotide match and mismatch values are 1 and -2, as this corresponds to 95% identity matches. In contrast, for BLASTN and DISCONTIGUOUS MEGABLAST, the values are 2 and -3 as they correspond to 85% identity [18].

Power users of BLAST often have a specially crafted set of command-line options that they find useful for their particular task. However, lacking a method to save these, they must write scripts or simply re-type them for each search. The BLAST+ applications can write the query, database, and command-line options for a BLAST search into a "strategy" file. A user may then rerun a set of commands by specifying the strategy file, though a new query and database can be specified with the command-line. This file is currently written as ASN.1 (Abstract Syntax Notation, a structured language similar to XML), but an XML option could be added in the future. Users can also upload this file to the NCBI BLAST web site to populate a BLAST search form, or download a strategy file for a search performed at the NCBI BLAST web site.

The BLAST+ applications have a number of new features. A GI or accession may be used as the query, with the actual sequence automatically retrieved from a BLAST database (the sequence must be available in a BLAST database) or from GenBank. The applications can send a search to NCBI servers as well as locally search a set of queries against a set of FASTA subject sequences [17].

Tables listing the command-line options, as well as their types and defaults, were provided as additional file 1 for this article.

### Database masking

Applying masking information to the BLAST database rather than the query will improve the workflow for BLAST users. A specialized tool, such as WindowMasker [19] or RepeatMasker [10], can provide masking information for a single-species database when it is created, and it becomes unnecessary to mask every query. Adding masking information to a BLAST database is a two step process. A file containing masking intervals in either XML or ASN.1 format is first produced, and then the information is added to the BLAST database. The NCBI C++ toolkit provides tools to produce this information for seg [20], dust [21], and WindowMasker [19]. Users may also provide intervals for algorithms not supported by the NCBI C++ toolkit; see the BLAST+ manual [17] for further information on how to produce a masked database. Currently, database masking is only available in soft-masking mode.

To test the performance of database masking, 163 human ESTs from UniGene cluster 235935 were searched against the build 36.1 reference assembly of the human genome [22]. RepeatMasker processed the EST queries, producing FASTA files with repeats identified in lower-case. RepeatMasker also processed the human genome FASTA files, locations of repeats were produced from that data, and those locations were then added as masking information to the BLAST database. Two sets of searches were run. One used the lower-case query masking to filter out interspersed repeats; the other used the database masking to do the same. Alignments with a score of 100 or more were retained. Table 1 presents the results, which indicate that differences in query masking with RepeatMasker caused extra matches. For example GI 14400848 is only 145 bases long and is not masked by RepeatMasker at all, but the portion of the genome it matches is masked. For GI 13529935 the last 78 bases are not masked, but the portion of the genome it matches is masked by RepeatMasker.

**Table 1:** Comparison of query versus database masking.

Type of masking	Number of alignments found	GIs of extra sequences found
Query	387	13529935, 14400848, 14430244, 14430457

Database	383	

Currently, database masking is not supported for searches of translated database sequences (i.e., tblastn and tblastx), but it will be supported in the near future.

Database masking is not a new concept. Kent [13] mentions cases where BLAT users might find repeat masking of the database useful. Morgulis et al. [23] also allow users to apply soft-masking to their database. In both of these cases, it is not simple to turn the masking on or off or to switch the type of masking (e.g., from RepeatMasker to WindowMasker). The implementation presented here allows this flexibility.

### Query splitting

Breaking longer queries into smaller pieces for processing can lead to significantly shorter search times. At the same time, splitting the query into pieces makes it possible to guarantee that the query length is always bounded, allowing the use of smaller data types in the lookup table. Use of smaller data types with a BLASTP search (protein-protein) shows no improvement for sequences under 500 residues, but performance increases by up to 2% as the sequence length increases to 8000 residues. Use of a smaller data type never makes performance worse, so it is used in the tests described in this section.

**Figure 2:**
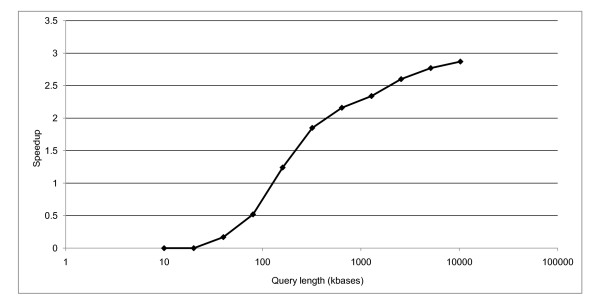
**Speedup of BLASTX searches for differently sized queries with and without query splitting**. Different sized pieces of [Genbank:NC_007113.2] were searched against a set of human proteins. The query length in kbases is on the x-axis, with a log scale. On the y-axis is the fractional speedup, which is defined as (T_baseline_/T_blastx_) - 1. Three searches were performed with both the baseline and the blastx applications (for each data point), and the lowest time for each application was used.

**Figure 3:**
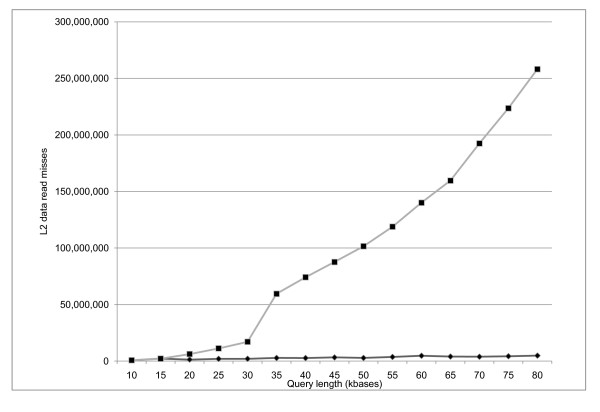
**L2 data cache misses for BLASTX searches with and without query splitting**. Cache misses were measured by Cachegrind [24] and only misses reading from the cache are shown. On the x-axis are different query lengths in kbases. The number of L2 cache misses is shown on the y-axis. The top line is for the baseline application without query splitting, the bottom line is for the blastx application. The queries are different sized pieces of [Genbank:NC_007113.2] searched against the set of human proteins used for Figure 2.

BLAST searches of differently-sized chunks of zebra fish chromosome 2 [Genbank:NC_007113.2] against a set of human proteins were performed to test the query splitting implementation. A baseline blastx application that does not split the query was prepared. Figure 2 presents the speedup for these searches, with speedup defined as (T_baseline_/T_blastx_) - 1. Query splitting decreases the search time for queries longer than 20 kbases, and the improvement continues with increasing query length. The Cachegrind memory profiling tool [24] confirmed a smaller number of cache misses with query splitting. Figure 3 presents those results. Figures 2 and 3 reflect an expect value cutoff of 1.0e-6.

Cameron et al. [14] replaced the BLAST lookup table with a DFA (Deterministic Finite Automaton) to improve the cache behavior. They reported a 10-15% reduction in search time for BLASTP (protein-protein) searches. Most proteins are too short to split, so no significant BLASTP improvements were apparent in the work presented here. This work emphasized improving the worst-case behavior typically seen with very long nucleotide queries. The query splitting approach does not preclude the use of a DFA or some other optimization instead of a lookup table.

### Partial subject sequence retrieval

**Figure 4:**
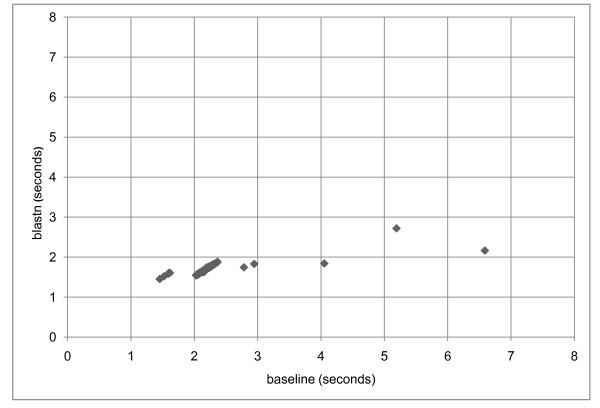
**Scatter plot of MEGABLAST search times with and without partial retrieval**. 163 human ESTs from UniGene cluster 235935 were searched against all human chromosomes [22]. On the x-axis are times for the baseline application; on the y-axis are times for the new blastn application. Sequences with the best improvement are those furthest to the right, and they also matched the largest number of subject sequences. A word size of 24 was used for the runs as well as database masking with RepeatMasker. Three searches were done with both the baseline and blastn application for each data point, and the lowest time for each application was used.

Partial retrieval of subject sequences is most effective when a small fraction of the subject sequence is required in the trace-back phase, such as in a search of ESTs against chromosomes. A baseline blastn application that retrieves the entire subject sequence in the trace-back phase was prepared. 163 human ESTs from UniGene cluster 235935 were searched against the masked human genome database from build 36.1 of the reference assembly [22]. Figure 4 presents search times with the standard blastn application and a baseline application. A word size of 24 and database masking (with RepeatMasker) was used. The ESTs with matches to the largest number of subject sequences showed the best improvement. The three rightmost data points on Figure 4 are for GIs 14429426, 13529935, and 34478925 (left to right). These three ESTs match four, six, and eight database sequences respectively. Overall, 158 sequences matched only one subject sequence, two matched two sequences and there was one match each for four, six, and eight sequences. As expected, performance did not improve for ESTs searched against a database of ESTs (data not shown).

### Retrieving subject sequences from an arbitrary source

An Abstract Data Type (ADT) supplies the subject sequences to be searched in the new BLAST code. This abstraction avoids coupling the BLAST engine to a particular database format. It permits a search of sequences in the "Short Read Archive" (SRA) at the NCBI through the SRA Software Development Kit [25]. An SRA BLAST web page accessible from the BLAST web site [11] was also created.

### Future development

Future developments include adding hard-masking support for databases, and making database masking available for programs with translated database sequences (tblastn and tblastx). At this point, only the scanning phase of the BLAST search is multi-threaded; we also plan to make the trace-back phase multi-threaded.

## Conclusions

We have reported on a new modular software library for BLAST. The design allows the addition of features that greatly benefit performance, such as query splitting and partial retrieval of subject sequences. It also allows the replacement of the lookup table with another design, so that new implementations can easily be added. An indexed version of MEGABLAST [23] was implemented using these libraries. The new library also supports a framework for retrieving subject sequences from arbitrary data sources. This framework, an Abstract Data Type (ADT), allows the use of different modules to read the BLAST databases in the NCBI C++ and the C toolkits. It is possible to write a new module to supply subject sequences to the BLAST engine using this ADT [16] without any modifications of the BLAST algorithm code. An ADT implementation has been written to support production searches of SRA sequences at the NCBI.

We also described a new set of BLAST command-line applications. The applications have a new, more logical organization that groups together similar types of searches in one application. The concept of a task allows a user to specify an optimal parameter set for a given task. Strategy files were also introduced, allowing a user to record parameters of a search in order to later rerun it in stand-alone mode or at the NCBI web site.

## Availability and requirements

BLAST is Public Domain software [26]. The latest version of BLAST can be retrieved from ftp://ftp.ncbi.nlm.nih.gov/blast/executables/blast+/LATEST. This software was implemented with the C and C++ programming languages and was tested under Microsoft Windows, Linux, and Mac OS X. There are no restrictions on use by non-academics. Query files and BLAST databases used for tests are available at ftp://ftp.ncbi.nih.gov/blast/demo/bmc.

## Authors' contributions

All authors participated in the design and coding of the software. TLM drafted the manuscript and the other authors provided feedback. All authors read and approved the final version of the manuscript.

## Supplementary Material

Additional file 1 **Eight tables list the command-line application options, as well as their types, default values, and a short explanation.** The first table has information common to the search applications blastn, blastp, blastx, tblastn, and tblastx. The next five tables describe options for those applications. The last two tables list the options for makeblastdb (used to build a blast database) and blastdbcmd (used to read a database). Click here for file
