# Supplementary material for: BLAST+: architecture and applications
Source: BMC Bioinformatics. 2009 Dec 15;10:421. doi: 10.1186/1471-2105-10-421 (PMC2803857; doi:10.1186/1471-2105-10-421)
Supplement: Additional file 1 — Eight tables list the command-line application options, as well as their types, default values, and a short explanation. The first table has information common to the search applications blastn, blastp, blastx, tblastn, and tblastx. The next five tables describe options for those applications. The last two tables list the options for makeblastdb (used to build a blast database) and blastdbcmd (used to read a database). [file 1471-2105-10-421-S1.PDF]

Table S1: Options common to all BLAST+ search applications. An option of type "flag" takes no argument, but if present is true. Some options are valid only for a local search ("remote" option not used), others are valid only for a remote search ("remote" option used).

option	**type**	**default value**	**description and notes**
db	string	none	BLAST database name.
query	string	stdin	Query file name.
query_loc	string	none	Location on the query sequence (Format: start-stop)
out	string	stdout	Output file name
evalue	real	10.0	Expect value (E) for saving hits
subject	string	none	File with subject sequence(s) to search.
subject_loc	string	none	Location on the subject sequence (Format: start-stop).
show_gis	flag	N/A	Show NCBI GIs in report.
num_descriptions	integer	500	Show one-line descriptions for this number of database sequences.
num_alignments	integer	250	Show alignments for this number of database sequences.
html	flag	N/A	Produce HTML output
gilist	string	none	Restrict search of database to GI's listed in this file. Local searches only
negative_gilist	string	none	Restrict search of database to everything except the GI's listed in this file. Local searches only.
entrez_query	string	none	Restrict search with the given Entrez query. Remote searches only.
culling_limit	integer	none	Delete a hit that is enveloped by at least this many higher-scoring hits.
best_hit_overhang	real	none	Best Hit algorithm overhang value (recommended value: 0.1)
best_hit_score_edge	real	none	Best Hit algorithm score edge value (recommended value: 0.1)
dbsize	integer	none	Effective size of the database
searchsp	integer	none	Effective length of the search space
import_search_strategy	string	none	Search strategy file to read.
export_search_strategy	string	none	Record search strategy to this file.
parse_deflines	flag	N/A	Parse query and subject bar delimited sequence identifiers (e.g., gi|129295).
num_threads	integer	1	Number of threads (CPUs) to use in blast search.
remote	flag	N/A	Execute search on NCBI servers?
oufmt	string	0	alignment view options:<br>0 = pairwise,<br>1 = query-anchored showing identities,<br>2 = query-anchored no identities,<br>3 = flat query-anchored, show identities,

			4 = flat query-anchored, no identities,<br>5 = XML Blast output,<br>6 = tabular,<br>7 = tabular with comment lines,<br>8 = Text ASN.1,<br>9 = Binary ASN.1<br>10 = Comma-separated values<br>Options 6, 7, and 10 can be additionally configured to produce a custom format specified by space delimited format specifiers.<br>The supported format specifiers are:<br>qseqid means Query Seq-id<br>qgi means Query GI<br>qacc means Query accesion<br>sseqid means Subject Seq-id<br>sallseqid means All subject Seq-id(s), separated by a ';'<br>sgi means Subject GI<br>sallgi means All subject GIs<br>sacc means Subject accession<br>sallacc means All subject accessions<br>qstart means Start of alignment in query<br>qend means End of alignment in query<br>sstart means Start of alignment in subject<br>send means End of alignment in subject<br>qseq means Aligned part of query sequence<br>sseq means Aligned part of subject sequence<br>evalue means Expect value<br>bitscore means Bit score<br>score means Raw score<br>length means Alignment length<br>pident means Percentage of identical matches<br>nident means Number of identical matches<br>mismatch means Number of mismatches<br>positive means Number of positive-scoring matches<br>gapopen means Number of gap openings<br>gaps means Total number of gap<br>ppos means Percentage of positive-scoring matches<br>frames means Query and subject frames separated by a '/'<br>qframe means Query frame<br>sframe means Subject frame<br>When not provided, the default value is:<br>'qseqid sseqid pident length mismatch gapopen qstart qend sstart send evalue bitscore', which is equivalent to the keyword 'std'

Table S2: Options for the blastn application. The blastn application searches a nucleotide query against nucleotide subject sequences or a nucleotide database. An option of type “flag” takes no arguments, but if present the argument is true. Options marked “experimental” may be removed or changed with little or no notice. Four different tasks are supported: 1.) “megablast”, for very similar sequences (e.g, sequencing errors), 2.) “dc-megablast”, typically used for inter-species comparisons, 3.) “blastn”, the traditional program used for inter-species comparisons, 4.) “blastn-short”, optimized for sequences less than 30 nucleotides.

**option**	**task(s)**	**type**	**default value**	**description and notes**
word_size	megablast	integer	28	Length of initial exact match.
word_size	dc-megablast	integer	11	Number of matching nucleotides in initial match. dc-megablast allows non-consecutive letters to match.
word_size	blastn	integer	11	Length of initial exact match.
word_size	blastn-short	integer	7	Length of initial exact match.
gapopen	megablast	integer	0	Cost to open a gap.
gapextend	megablast	integer	none	Cost to extend a gap. This default is a function of reward/penalty value.
gapopen	blastn, blastn-short, dc-megablast	integer	5	Cost to open a gap.
gapextend	blastn, blastn-short, dc-megablast	integer	2	Cost to extend a gap.
reward	megablast	integer	1	Reward for a nucleotide match.
penalty	megablast	integer	-2	Penalty for a nucleotide mismatch.
reward	blastn, dc-megablast	integer	2	Reward for a nucleotide match.
penalty	blastn, dc-megablast	integer	-3	Penalty for a nucleotide mismatch.
reward	blastn-short	integer	1	Reward for a nucleotide match.

penalty	blastn-short	integer	-3	Penalty for a nucleotide mismatch.
strand	all	string	both	Query strand(s) to search against database/subject. Choice of both, minus, or plus.
dust	all	string	20 64 1	Filter query sequence with dust.
filtering_db	all	string	none	Mask query using the sequences in this database.
window_masker_taxid	all	integer	none	Enable WindowMasker filtering using a Taxonomic ID.<br>NOTE: experimental.
window_masker_db	all	string	none	Enable WindowMasker filtering using this file.<br>NOTE: experimental.
soft_masking	all	boolean	true	Apply filtering locations as soft masks.
lcase_masking	all	flag	N/A	Use lower case filtering in query and subject sequence(s)?
db_soft_masking	all	integer	none	Filtering algorithm ID to apply to the BLAST database as soft masking.
perc_identity	all	integer	0	Percent identity cutoff.
template_type	dc-megablast	string	coding	Discontiguous MegaBLAST template type. Allowed values are coding, optimal and coding_and_optimal.
template_length	dc-megablast	integer	18	Discontiguous MegaBLAST template length.
use_index	megablast	boolean	false	Use MegaBLAST database index.
index_name	megablast	string	none	MegaBLAST database index name.
xdrop_ungap	all	real	20	Heuristic value (in bits) for ungapped extensions.
xdrop_gap	all	real	30	Heuristic value (in bits) for preliminary gapped extensions.
xdrop_gap_final	all	real	100	Heuristic value (in bits) for final gapped alignment.
no_greedy	megablast	flag	N/A	Use non-greedy dynamic programming extension.
min_raw_gapped_score	all	integer	none	Minimum raw gapped score to keep an alignment in the preliminary gapped and trace-back stages. Normally set based

				upon expect value.
ungapped	all	flag	N/A	Perform ungapped alignment.
window_size	dc-megablast	integer	40	Multiple hits window size, use 0 to specify 1-hit algorithm

Table S3: Options for the blastp application. The blastp application searches a protein sequence against protein subject sequences or a protein database. An option of type "flag" takes no arguments, but if present the argument is true. Two different tasks are supported: 1.) "blastp", for standard protein-protein comparisons, 2.) "blastp-short", optimized for query sequences shorter than 30 residues. This table reflects the 2.2.23 BLAST+ release. On earlier releases the blastp-short task was not implemented.

**option**	**task**	**type**	**default value**	**description and notes**
word_size	blastp	integer	3	Word size of initial match.
word_size	blastp-short	integer	2	Word size of initial match.
gapopen	blastp	integer	11	Cost to open a gap.
gapextend	blastp	integer	1	Cost to extend a gap.
gapopen	blastp-short	integer	9	Cost to open a gap.
gapextend	blastp-short	integer	1	Cost to extend a gap.
matrix	blastp	string	BLOSUM62	Scoring matrix name.
matrix	blastp-short	string	PAM30	Scoring matrix name.
threshold	blastp	integer	11	Minimum score to add a word to the BLAST lookup table.
threshold	blastp-short	integer	16	Minimum score to add a word to the BLAST lookup table.
comp_based_stats	blastp	string	2	Use composition-based statistics:<br>D or d: default (equivalent to 2)<br>0 or F or f: no composition-based statistics<br>1: Composition-based statistics as in NAR 29:2994-3005, 2001<br>2 or T or t : Composition-based score adjustment as in Bioinformatics

				21:902-911, 2005, conditioned on sequence properties<br>3: Composition-based score adjustment as in Bioinformatics 21:902-911, 2005, unconditionally
comp_based_stats	blastp-short	string	0	Use composition-based statistics :<br>D or d: default (equivalent to 2)<br>0 or F or f: no composition-based statistics<br>1: Composition-based statistics as in NAR 29:2994-3005, 2001<br>2 or T or t : Composition-based score adjustment as in Bioinformatics 21:902-911, 2005, conditioned on sequence properties<br>3: Composition-based score adjustment as in Bioinformatics 21:902-911, 2005, unconditionally
seg	all	string	no	Filter query sequence with SEG (Format: 'yes', 'window locut hicut', or 'no' to disable).
soft_masking	blastp	boolean	false	Apply filtering locations as soft masks
xdrop_ungap	all	real	7	Heuristic value (in bits) for ungapped extensions
xdrop_gap	all	real	15	Heuristic value (in bits) for preliminary gapped extensions.
xdrop_gap_final	all	real	25	Heuristic value (in bits) for final gapped alignment/
window_size	blastp	integer	40	Multiple hits window size, use 0 to specify 1-hit algorithm.
window_size	blastp-short	integer	15	Multiple hits window size, use 0 to specify 1-hit algorithm.
use_sw_tback	all	flag	N/A	Compute locally optimal Smith-Waterman alignments?

Table S4: Options for the blastx application. The blastx application translates a nucleotide query and searches it against protein subject sequences or a protein database.

option	type	default value	description and notes
word_size	integer	3	Word size for initial match.
gapopen	integer	11	Cost to open a gap.
gapextend	integer	1	Cost to extend a gap.
matrix	string	BLOSUM62	Scoring matrix name.
threshold	integer	12	Minimum score to add a word to the BLAST lookup table.
seg	string	12 2.2 2.5	Filter query sequence with SEG (Format: 'yes', 'window locut hicut', or 'no' to disable).
soft_masking	boolean	false	Apply filtering locations as soft masks.
xdrop_ungap	real	7	Heuristic value (in bits) for ungapped extensions.
xdrop_gap	real	15	Heuristic value (in bits) for preliminary gapped extensions.
xdrop_gap_final	real	25	Heuristic value (in bits) for final gapped alignment.
window_size	integer	40	Multiple hits window size, use 0 to specify 1-hit algorithm.
strand	string	both	Query strand(s) to search against database/subject. Choice of both, minus, or plus.
query_genetic_code	integer	1	Genetic code to translate query, see ftp://ftp.ncbi.nih.gov/entrez/misc/data/gc.prt
frame_shift_penalty	integer	0	Frame shift penalty (for use with out-of-frame gapped alignment).<br>NOTE: statistics may not be correct with the option
max_intron_length	integer	0	Length of the largest intron allowed in a translated nucleotide sequence when linking multiple distinct alignments (a negative value disables linking).

Table S5: Options for the tblastn application. The tblastn application searches a protein query against nucleotide subject sequences or a nucleotide database translated at search time.

**option**	**type**	**default value**	**description and notes**
word_size	integer	3	Word size for initial match.
gapopen	integer	11	Cost to open a gap.
gapextend	integer	1	Cost to extend a gap.
matrix	string	BLOSUM62	Scoring matrix name.
threshold	integer	13	Minimum score to add a word to the BLAST lookup table.
seg	string	12 2.2 2.5	Filter query sequence with SEG (Format: 'yes', 'window locut hicut', or 'no' to disable).
soft_masking	boolean	false	Apply filtering locations as soft masks.
xdrop_ungap	real	7	Heuristic value (in bits) for ungapped extensions.
xdrop_gap	real	15	Heuristic value (in bits) for preliminary gapped extensions.
xdrop_gap_final	real	25	Heuristic value (in bits) for final gapped alignment.
window_size	integer	40	Multiple hits window size, use 0 to specify 1-hit algorithm.
db_gen_code	integer	1	Genetic code to translate subject sequences, see ftp://ftp.ncbi.nih.gov/entrez/misc/data/gc.prt
frame_shift_penalty	integer	0	Frame shift penalty (for use with out-of-frame gapped alignment.<br>NOTE: statistics may not be correct with the option
max_intron_length	integer	0	Length of the largest intron allowed in a translated nucleotide sequence when linking multiple distinct alignments (a negative value disables linking).
comp_based_stats	string	D	Use composition-based statistics for tblastn:<br>D or d: default (equivalent to 2)<br>0 or F or f: no composition-based statistics<br>1: Composition-based statistics as in NAR 29:2994-3005, 2001<br>2 or T or t : Composition-based score adjustment as in Bioinformatics<br>21:902-911, 2005, conditioned on sequence properties<br>3: Composition-based score adjustment as in Bioinformatics 21:902-911, 2005, unconditionally<br>Default = `2'

Table S6: Options for the tblastx application. The tblastx application searches a translated nucleotide query against translated nucleotide subject sequences or a translated nucleotide database An option of type “flag” takes no arguments, but if present the argument is true. This table reflects the 2.2.23 BLAST+ release.

**option**	**type**	**default value**	**description and notes**
word_size	integer	3	Word size for initial match.
matrix	string	BLOSUM62	Scoring matrix name.
threshold	integer	13	Minimum word score to add the word to the BLAST lookup table.
seg	string	12 2.2 2.5	Filter query sequence with SEG (Format: 'yes', 'window locut hicut', or 'no' to disable).
soft_masking	boolean	false	Apply filtering locations as soft masks.
xdrop_ungap	real	7	Heuristic value (in bits) for ungapped extensions.
window_size	integer	40	Multiple hits window size, use 0 to specify 1-hit algorithm.
strand	string	both	Query strand(s) to search against database subject sequences. Choice of both, minus, or plus.
query_genetic_code	integer	1	Genetic code to translate query, see ftp://ftp.ncbi.nih.gov/entrez/misc/data/gc.prt
db_gen_code	integer	1	Genetic code to translate subject sequences, see ftp://ftp.ncbi.nih.gov/entrez/misc/data/gc.prt
max_intron_length	integer	0	Length of the largest intron allowed in a translated nucleotide sequence when linking multiple distinct alignments (a negative value disables linking)

Table S7: Options for the makeblastdb application. This application builds a BLAST database. An option of type "flag" takes no arguments, but if present the argument is true.

option	type	default value	description
in	string	stdin	Input file/database name; the data type is automatically detected, it may be any of the following:<br>FASTA file(s) and/or<br>BLAST database(s)
dbtype	string	prot	Molecule type of input, values can be nucl or prot.
title	string	none	Title for BLAST database. If not set the input file name will be used.
parse_seqids	flag	N/A	Parse bar delimited sequence identifiers (e.g., gi|129295) in FASTA input.
hash_index	flag	N/A	Create index of sequence hash values.
mask_data	string	none	Comma-separated list of input files containing masking data as produced by NCBI masking applications (e.g. dustmasker, segmasker, windowmasker).
out	string	input file name	Name of BLAST database to be created. Input file name is used if none provided. This field is required if input consists of multiple files.
max_file_size	string	1GB	Maximum file size to use for BLAST database.
taxid	integer	none	Taxonomy ID to assign to all sequences.
taxid_map	string	none	File mapping sequence IDs to taxonomy IDs.
logfile	string	none	Program log file (default is stderr).

Table S8: Options for blastdbcmd application. This application reads a BLAST database and produces reports.

option	type	default value	description and notes
db	string	nr	BLAST database name.
dbtype	string	guess	Molecule type stored in BLAST database, one of nucl, prot, or guess.
entry	string	none	Comma-delimited search string(s) of sequence identifiers: e.g.: 555, AC147927, 'gnl|dbname|tag', or 'all' to select all sequences in the database
entry_batch	string	none	Input file for batch processing (Format: one entry per line)
pig	integer	none	PIG (protein identity group) to retrieve.
info	flag	N/A	Print BLAST database information.
range	string	none	Range of sequence to extract (Format: start-stop).
strand	string	plus	Strand of nucleotide sequence to extract. Choice of plus or minus.
mask_sequence_with	string	none	Produce lower-case masked FASTA using the algorithm IDs specified.
out	string	stdout	Output file name.
outfmt	string	%f	Output format, where the available format specifiers are:<br>%f means sequence in FASTA format<br>%s means sequence data (without defline)<br>%a means accession<br>%g means gi<br>%o means ordinal id (OID)<br>%t means sequence title<br>%l means sequence length<br>%T means taxid<br>%L means common taxonomic name<br>%S means scientific name<br>%P means PIG<br>%mX means sequence masking data, where X is an optional comma-separated list of integers to specify the algorithm ID(s) to display (or all masks if absent or invalid specification). Masking data will be

			displayed as a series of 'N-M' values separated by ';' or the word 'none' if none are available. For every format except '%f', each line of output will correspond to a sequence.
target_only	flag	N/A	Definition line should contain target GI only.
get_dups	flag	N/A	Retrieve duplicate accessions.
line_length	integer	80	Line length for output.
ctrl_a	flag	N/A	Use Ctrl-A as the non-redundant definition line separator.
